# Supplementary material for: Chromothripsis during telomere crisis is independent of NHEJ, and consistent with a replicative origin
Source: Genome Res. 2019 May;29(5):737–49. doi: 10.1101/gr.240705.118 (PMC6499312; doi:10.1101/gr.240705.118)
Supplement: Supplemental Material [file supp_gr.240705.118_Supplemental_file_1.zip › contigs/annotated_contigs/DB93/contig.2.DB93_length_657_mean_cov_7.43835616438.docx]

**DB93_length_657_mean_cov_7.43835616438**

```
TTTTATCTCAATGCATGCAGGAAAGAGGGAAATGTTCTCCATAGGTTTAGAGACTTGTAGGCATTAGAATCCCTGTTAAGACTTGTTCT
 >chr3:116460853-116461206 + E=7e-196
TTGAAAGGCAAACCTTCAACCCTAAAATCTAACTCAAACCTTGAATGGAAGGAATAAGCCTCTGCATTCATCACTAAGGCAAGTAGTGT

GCCCCAAAGCCTGGCCAGGGCAATAACTTATGAGCAAGTTAGAGCTGAGGATATCAAGTCAAATAACCAAGACCGCATTGTGAGAAGAA

AAAAAAAAGGGGGGAGAGAGAGAGAGAGATCTGGAGTTTAGCAGGGACTCTGAGGACAGCTGCAGACTGCCAGACAAGAATTA|AA|TT
                                                                                    >ch
CCACTGGGTCAGATAAAGCAATATAATTCTAATGTTATTTGCCTTAAGTCCAGAGGAGTGATAGGATTGGTTAGGTGACATTTTGTTGA
r3:116461482-116461789 - E=6e-173
AGACAGAGTAGGAGAAATGTTTGGGGGAAAAAATTCCCGATATGGCATAATATCATAATGTCCACTCATAATTGCTAAATGACAATTGT

CATTTGCTTTTATATCAGATATCTAATTAATCAAACTTTCTGGTTAAGTGCCTCCAATTATTAACAATCCTGCTCAGAGTTAAGATTAG

CATCCAAGGATTTTCCTCCATATTTCTTTTCAGAGG
```
